# Optimizing access to and use of formal dementia care: Qualitative findings from the European Actifcare study

**DOI:** 10.1111/hsc.12804

**Published:** 2019-07-10

**Authors:** Liselot Kerpershoek, Claire Wolfs, Frans Verhey, Hannah Jelley, Bob Woods, Anja Bieber, Gabriele Bartoszek, Astrid Stephan, Geir Selbaek, Siren Eriksen, Britt‐Marie Sjölund, Louise Hopper, Kate Irving, Maria J. Marques, Manuel Gonçalves‐Pereira, Daniel Portolani, Orazio Zanetti, Marjolein Vugt, The Actifcare Consortium

**Affiliations:** ^1^ Alzheimer Centre Limburg Maastricht University Maastricht Netherlands; ^2^ Bangor University Bangor UK; ^3^ Martin‐Luther University Halle‐Wittenberg Halle Germany; ^4^ Norwegian National Advisory Unit on Ageing and Health Vestfold Hospital Tonsberg Norway; ^5^ Centre for Old Age Psychiatry Research Innlandet Hospital Trust Ottestad Norway; ^6^ Faculty of Medicine University of Oslo Oslo Norway; ^7^ Aging Research Center (ARC), Department of Neurobiology, Care Sciences and Society (NVS) Karolinska Institutet and Stockholm University Stockholm Sweden; ^8^ Faculty of Health and Occupational Studies, Department of Health and Caring Sciences University of Gävle Gävle Sweden; ^9^ School of Nursing and Human Sciences Dublin City University Dublin Ireland; ^10^ CEDOC, Nova Medical School, Faculdade de Ciências Médicas Universidade Nova de Lisboa Lisbon Portugal; ^11^ IRCCS Istituto Centro San Giovanni di Dio Fatebenefratelli Brescia Italy

**Keywords:** access to care, dementia, in‐depth interviews, informal care, service use

## Abstract

This paper reports on qualitative data from the Actifcare study investigating experiences, attitudes, barriers and facilitators concerning access to and use of formal care. A total of 85 semi‐structured in‐depth interviews were conducted in eight European countries. Results were analysed with a deductive content analysis, first within country and then integrated in a cross‐national analysis. Overall, analysis of the in‐depth interviews revealed two major themes with five subcategories. The results can be summarised in an optimal pathway for access to dementia care. This pathway includes fixed factors such as disease‐related factors and system‐related factors. In addition there are personal factors that are subject to change such as attitudes towards care. An important finding consisted of the necessity of having sufficient information about the disease and available care and having a key contact person to guide you through the process of finding suitable care while monitoring your needs. In addition, it is important to involve your social network as they can take on care‐giving tasks. It is helpful to have a diagnosis (in most countries). Concerning decision‐making, the person closest to the person with dementia is in the majority of cases the one who makes the ultimate decision to access and use services and he/she should therefore be supported in this process. These results provide insight into the factors that influence the pathway to formal care use and help professionals to enhance access to formal dementia care by focusing on factors that can be modified.

**What is known about this topic?**

- People with dementia and their informal carers often do not use the amount and type of services that they objectively need
- People trying to access formal care experience this process as difficult and time‐consuming

**What this paper adds**

- There are substantial differences between European countries in access to and use of formal care services
- An appointed key contact person is crucial for optimising access to and the use of dementia care
- A solid social network can postpone the use of formal dementia care
- People with dementia and their informal carers should be supported by healthcare professionals in decision‐making concerning care, and healthcare professionals should be trained in this support.

## 1 INTRODUCTION

Dementia is a progressive syndrome, with symptoms affecting cognition, behaviour and the ability to carry out activities of daily living. As the disease progresses, an increasing amount of care is needed. Nowadays, the use of home care for people with dementia is encouraged. In an ideal situation for society, needs would first be covered by informal care, until formal community services become necessary (Jiménez‐Martín & Prieto, 2012), and it will then complement informal care. There are several services that can be offered at home, such as help with personal care, day care or nursing care (so‐called formal care).

Previous research has shown that people often do not use the amount and type of services that they objectively need (Phillipson, Jones, & Magee, 2014). Different barriers have been described, but peculiarly, many carers mention that they do not use services because they simply feel it is not necessary (Carpentier, Ducharme, Kergoat, & Bergman, 2008). This is often regretted in later stages, where carers indicate that they would now prefer to have used services in an earlier stage, also known as the early stage needs paradox (Boots, Wolfs, Verhey, Kempen, & de Vugt, 2015). Another reason for non‐use is experiencing difficulties in accessing suitable services. People trying to access formal care experience this process as difficult and time‐consuming (Peel & Harding, 2014). Informal carers express the need for better advice and support in this process of accessing formal care. In a society where it is encouraged to live in the community as long as possible, it is important that there are as few barriers as possible in accessing care.

### 1.1 The Actifcare study

**Figure 1:**
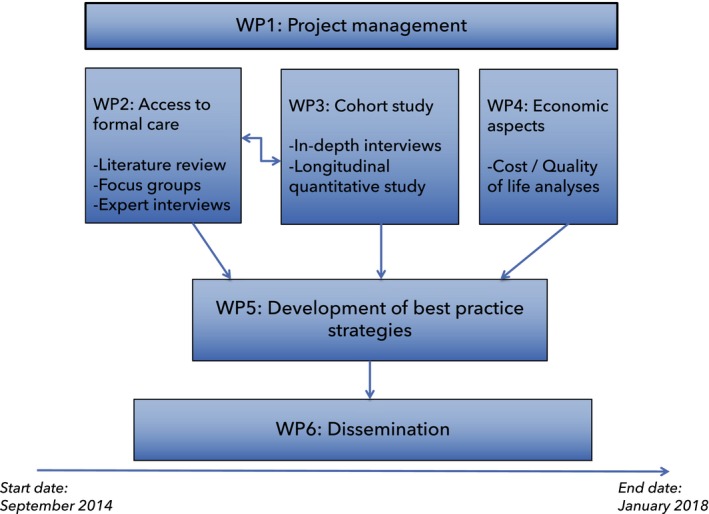
The design of the Actifcare study

This interview study is part of the larger Actifcare study (Kerpershoek et al., 2016) which comprises of several work packages (see Figure 1) in which access to and the use of formal care was explored in eight European countries (the Netherlands (NL), Germany (DE), United Kingdom (UK), Ireland (IE), Sweden (SE), Norway (NO), Portugal (PT), Italy (IT)). A mapping system was used to provide an overview of the structures and services for those with dementia (Bieber et al., 2018). A range of 29 different services for people with dementia and their carers were identified and compared across the countries (Kerpershoek et al., 2016).

In all of these countries, the general practitioner (GP) is the first professional to turn to. In some countries, GP’s establish the dementia diagnosis (SE, NL, NO, partially in PT), while in the other countries the GP usually refers to a specialist for diagnosis.

After receiving the diagnosis, people might be referred to other dementia services such as Community Mental Health Teams in NL and UK or the Local Authority dementia teams in NO. In Italy, there is a large group of people who hire privately paid migrants to provide care at home. Importantly, not all countries have post‐diagnostic pathways. Existing pathways are described in detail in a different Actifcare paper (Bieber et al., 2018).

We aim to provide a more detailed and in‐depth insight into people's experiences, attitudes, barriers and facilitators towards access to care in an explorative manner. Accordingly, we aim to describe in more detail the optimal circumstances for timely access to care.

Research questions:

- What are the experiences and attitudes of people with dementia and their informal carers concerning accessing and using formal care?
- Which barriers and facilitators are reported concerning the access to and use of formal care?
- What would be the optimal pathway to formal dementia care?

## 2 METHODS

The Actifcare study (ACcess to TImely Formal Care), a JPND‐funded project, focused on access to home‐ and community‐based dementia care, for people in the middle stages of dementia. Various research methods were used, such as literature reviews, focus groups, expert interviews, cost‐consequence analyses and a cohort study (Kerpershoek et al., 2016) (see Figure 1). The current individual interviews with people with dementia and their informal carers build on the results of the cohort study and of the focus groups (Stephan et al., 2018). In the quantitative part of the study, data were collected about service use, needs, quality of life and various other variables at baseline, six months and twelve months follow‐up.

### 2.1 Study participants

The Actifcare cohort consists of 451 community‐dwelling dyads of people with middle‐stage dementia (Kerpershoek et al., 2016) (mean age: 77.8, mean Mini Mental State Examination (MMSE) score: 19 and their informal carers (mean age: 66.4). The latter could be a spouse, family member or friend. At baseline, the dyads did not use formal care, but they were expected, based on clinical judgement, to commence within the next year. The complete inclusion and exclusion criteria and the recruitment method are described in the design paper (Kerpershoek et al., 2016). The definition of formal care used in Actifcare includes home nursing care, day care service, community or long‐term medical care, nursing and social care structures. It excludes domestic home help, housekeepers, volunteers, support groups, transport services and meal programmes. These services were selected as we are particularly interested in access to dementia‐specific services, rather than general services for the elderly people.

In each country, the research group was asked to select ten dyads from the Actifcare cohort. Only those people with dementia who were able to communicate and give retrospective opinions participated. The dyad was interviewed either separately or together, depending on the cognitive abilities and the wishes of the person with dementia. A purposive sampling selection procedure was used, to ensure a diverse sample regarding care use, gender, age and education. Half of the sample represented people who started using formal care, and half of the sample had not yet started to use formal care. Interviewing both these groups allowed us to include a broad range of attitudes, opinions and experiences.

### 2.2 Procedure

Written informed consent was signed by the dyad according to the ethical procedures in each country. The semi‐structured interviews were conducted either at the researchers' site or at the participants' home. All interviews were audio recorded and transcribed verbatim for analysis purposes. Interviewers were members of the Actifcare research group of each country, who were acquainted with the participants due to previous assessments in the cohort study. Their backgrounds include research nurses, psychologists and physicians.

### 2.3 Interview guide

In‐depth interviews were selected as this allowed us to get a broader insight into people's motives and attitudes to supplement the quantitative information from the Actifcare cohort study. The interview guide (see Appendix ) was built upon the outcomes of the focus groups conducted earlier in the Actifcare project (Stephan et al., 2018 in peer review). The interview topics were attitudes towards services, care and personal experienced facilitators and barriers to access. Specific questions were formulated to examine 'Receiving the diagnosis', 'Attitudes towards formal care', 'Tension between independence and acceptance of care', 'Exchanging views within the family', and 'Cooperation with healthcare professionals'.

### 2.4 Data analysis

First, each country transcribed their own interviews verbatim and analysed them following a deductive qualitative content analysis method following the exact same protocol. Each country reported their themes, categories and quotes. These findings were reported in a narrative and comprehensive way and translated to English. Second, two members of the Dutch research group carried out a cross‐national comparison of these translated documents, to reveal differences and similarities across the findings while constantly comparing. Afterward, these two members compared and synthesised their findings. Each country then carefully checked the analysis to guarantee that no information was misinterpreted. Throughout this process the research groups collaborated closely.

## 3 RESULTS

### 3.1 Participants

A total of 85 in‐depth interviews were conducted between January and July 2016 (see Table 1). In the majority of the interviews informal carers participated on their own; in some of the interviews, the person with dementia also participated, depending on their own wish. Some people with dementia were also interviewed on their own. The interviews lasted on average 35.8 min. The mean age of the interviewed people with dementia was 79.1 and of the informal carers 66.2. Of the people with dementia, 44% were male, while 29% of the informal carers were male. The dyad relations were as follows: 61% spouse or partner, 35% child and 4% other relatives.

**Table 1:** Number of interviews: some dyads were interviewed simultaneously, and others separately

Country	NL	DE	UK	SE	NO	IE	IT	PT
Total # dyads	10	11	10	10	10	10	12	12
Dyadic interviews	8	–	7	10	8	–	1	8
Interviews with PwD only	–	–	–	–	5	10	8	2
Interviews with carer only	2	11	3	10	6	10	11	4

In the following paragraphs, the categories are described in‐depth with accompanying illustrating quotes. Each quote is labelled with a code referring to the country (NL = the Netherlands, DE = Germany, UK = United Kingdom, IE = Ireland, NW = Norway, SE = Sweden, PT = Portugal, IT = Italy), and it states whether the quote derived from a (non)‐formal care user.

**Theme 1)** Conditions to enhance access:

1. Personal.
2. Diagnosis & post‐diagnostic support.
3. System/process.

**Theme 2)** Decision‐making.

1. Decisional conflicts.
2. Involvement of others.

**Figure 2:**
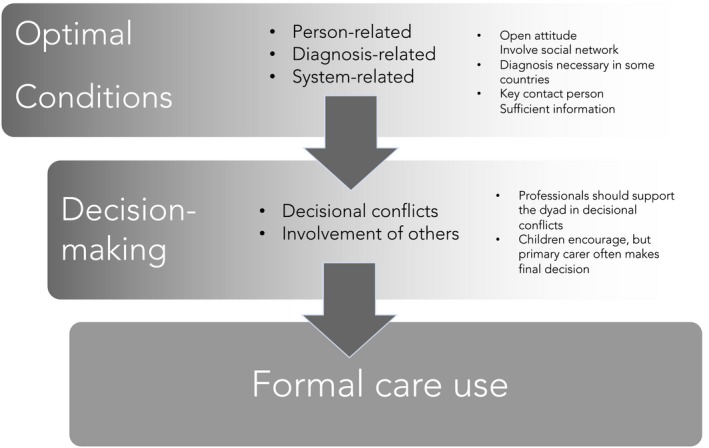
An optimal pathway for finding access to care

To answer the research question “What would be the optimal pathway to formal dementia care?” we summarised the findings in Figure 2. Analysis of the in‐depth interviews revealed two major themes with five subcategories. These categories are visualised in a pathway showing optimal access to care. Overall, there are fixed factors that cannot be changed, such as disease‐related factors and system‐related factors. In addition, there are personal factors that are subject to change such as attitudes. To enhance optimal access to care, the most important factors are described below.

#### 3.1.1 Theme 1) Conditions to enhance access: Personal factors

##### Attitude and need for care

The majority of the carers reported having an open attitude towards receiving care, as long as the request for care is 'justified', and care is appropriate for the perceived need (IT, NL). However, in Portugal the presence of stigma was reported by participants who received little information about what dementia entails. This resulted in reluctance in accessing formal care. Having an open attitude functions as a facilitator in accessing formal care. Carers reported that a good insight into the disease fosters an open attitude. There might be some initial embarrassment in relation to personal care, which was mainly related to the influence on privacy. Only a few participants reported a feeling of shame.

“It is a privilege; I see it as a privilege that you can receive care. You have to accept it in my opinion and don't think: I can do this all on my own, because at some point you cannot do that anymore. It might work for two weeks, but no longer”. (NL1002, Formal care use, Carer, )

Using a certain type of care might make it easier to accept other types of care, as a gradual build‐up is reported as helpful. In Sweden, it was revealed, for instance, that if people made use of meals on wheels services, the threshold to start using personal care was lowered.

In the post‐diagnostic period carers felt that accepting services at an early stage would have a negative impact on the person with dementia's independence and self‐esteem. However, independence might also be enhanced because of socialisation and increased motivation through engagement in activities. For carers, the use of formal care could enhance their independence, giving more time for their personal activities and social life.

Besides (changes in) attitudes, other reasons to start using formal care were reported in relation to an increase in disease severity, occurrence of crisis situation or alleviating strain of the carer.

*“*What I think is important is that the request is justified, I have a reason to ask for this, if there is the chance to have it, it is welcomed”. (IT8047, Formal care use, Person with dementia)

In the southern countries (PT and IT), there was a sense of moral obligation towards informal care‐giving. The role of the family in care‐giving is of vital importance. The fact that formal care is also expensive, further contributes to this. Other reasons to postpone the use of care were the person with dementia refusing formal care, not being emotionally ready as a carer, or experiencing a sense of guilt or shame towards the person being cared for. The main reason put forward by carers was that they try to maintain autonomy and do not want to resign their care tasks (IE, NL, NO, SE, PT). Some carers indicated that only now that care was in place, they realised how they themselves were struggling to manage caring for the person even when they had initially felt reluctant to accept support. Another reason to postpone care was the availability of a large social network and therefore plenty of people to turn to for help (IE, NL). Several carers underscore that because of their good health and ability to help they did not need assistance yet.

“ I'd like to do it alone for as long as I can. This is important for my husband too, because I’m the key contact person for him”. (DE2043, No formal care use, Carer)

#### 3.1.2 Theme 1) Conditions to enhance access: Diagnosis & post‐diagnostic support

Overall, there was ambiguity concerning this topic within and between countries. Some carers said that it is not necessary to have a diagnosis to access care (DE, NO, NL, SE, IT). In PT, one carer even suggested that having a diagnosis would impede access to day care or nursing homes.

“I felt that the diagnosis would have been an exclusion criterion”. (PT7023, Formal care use, Carer)

Some carers thought that a diagnosis would be a precondition for receiving care (IT, DE, IE, NO, SE). Even if it might not be a precondition, it served sometimes as a facilitator, as it provides you with an incentive to look for help.

More specifically, differences were reported between countries concerning the role of the general practitioner (GP). In IT and PT, GP’s are highly esteemed, but there was a sense of disappointment in their lack of diagnostic experience and knowledge of available services. GP’s perceive the diagnosis as a specialists’ domain. This resulted in a long process before a diagnosis was stated. The same was reported in NO, where several had asked for assessment but this was not provided.

In some countries, carers mentioned that care was offered immediately after the diagnosis (NL, NO, UK), in either a direct way or an indirect way in offering help when this will be necessary*.* Carers indicated that good information post‐diagnosis could postpone the need for care, but in general neither information nor care was offered in a structured way. Some participants stated that they were guided towards post‐ diagnostic care by health and social care professionals. Other participants indicated that they did not receive any practical advice following the diagnosis that would have helped them, and reported that advice was now merely focused on medical needs (IE, NL, IT, NO, PT).

“In this disease, no one cares to support the family. The carer does not exist. The neurologist only told me that this tends to get worse and worse [referring to the dementia progression]. Besides medication, the doctor said there was nothing else to do (…).” (IT8043, No formal care use, Carer)

“Nobody sort of sat you down and said, “Well this is what's going to happen. No, I didn't find him (GP) helpful.'' (IE6015, No formal care use, Carer)

#### 3.1.3 Theme 1) Conditions to enhance access: System‐related factors

### 3.2 Barriers and facilitators

The most often reported barrier across countries was a lack of knowledge and information. Adequate information about dementia and about available resources is necessary. Carers indicated that it is essential that you search pro‐actively yourself if insufficient information is provided.

“I missed a list where I could find exactly written which services I could have had access to”. (IT8029, Formal care use, Carer)

“There would be a huge crowd of us that would much prefer to see a simple little leaflet put in the bag and find it there that night, not think well I must turn on the computer and remember what button to press to get into dementia.” (IE6016, No formal care use, Person with dementia)

In addition, waiting lists were a significant barrier. In PT, carers reported having to wait for months before obtaining access to services. To avoid this, some carers had to contact private services.

I think the access to formal care is very similar to the process of getting a job. If we do not have a friend in the system, we need to wait a long time.' (PT7032. Formal care use, Carer.

Having an assigned key contact person who you can approach for questions and concerns was mentioned as an important facilitator. It was reported that it is confusing if there are too many different professionals involved, and that knowing whom to turn to is a relief.

“These people keep coming and ringing up‐ I'm getting confused. So many people ringing you, I get confused about who I'm talking to sometimes”. (UK2023, Formal care use, Carer)

Other important characteristics of healthcare professionals were that they should be easy to reach and have dementia‐specific skills and knowledge. Where they do not have the skills or knowledge to cope with a certain situation, it is important that they are able to refer adequately.

If services are not tailored to the individual's situation, this is experienced as a barrier in some countries and could lead to rejection of further use of formal care. Everyone has different backgrounds and individual preferences so for example day care programmes that are well suited for older people may not be suited for people with young onset dementia.

“Poor [other person with dementia attending the group] next to me could not remember where he left his phone. Do you understand? The lady across the table, because of her memory going crazy, she was suffering from severe anxiety but it was one package fits all and it does not” (Formal care use, Person with dementia, IE6002)

More explicitly, the preference for tailor‐made services was expressed in those countries where formal care was already well in place. This is in contrast with the southern countries, where this wish was not mentioned specifically, as the wish for affordable, well‐organised and dementia‐specific care has the priority.

Carers from the UK indicated that it is helpful to have a sense of control over the timing and nature of the help you receive, for example being able to indicate the timeslots during the week that are best suited. Another reported barrier in some countries was cost of services: a lack of financial support would lead to non‐use of services (PT, IE). In IE, access to care was hindered for those who were not eligible for a state‐sponsored medical card. In addition, carers mentioned that the ability to turn to private care instead of public, which was more expensive, would lead to quicker help. People are often entitled to receiving financial compensation for services but they are unaware of this (NL, DE, UK, SE).

“We did not access other care services besides day centre because it was very expensive”. (PT7039, Formal care use, Carer)

#### 3.2.1 THEME 2) Decision‐making concerning formal care use: Decisional conflicts

The majority of carers indicated that the involvement of the person with dementia in the decision‐making process concerning care use depends on levels of awareness of difficulties. If the person with dementia lacked awareness, it was difficult for the carer to ignore their reluctance: most carers indicated they would consult with the person with dementia and never force them to accept an offer of provision of formal care if they did not want to.

“They [family members] make the choice, ah yeah…At least they know what they are talking about, but I might not” (IE6017, Formal care use, Person with dementia)

Care‐givers often experienced difficulties in exchanging views with the person with dementia on care needs due to communication difficulties. The previously discussed cultural difference between the southern countries (IT and PT) where there is a sense of moral obligation towards care‐giving also relates to decision‐making, to the extent that it is more a family decision than a carer decision only.

#### 3.2.2 Theme 2) Decision‐making: Involvement of others

For a significant number of participating dyads, other family members are involved in the decision‐making process. Children are often the ones who encourage their parents to look for help in the first place and offer emotional and practical support (NL, SE, DE). Formal care use is facilitated when children who are consulted have similar views regarding care. Decisions to take up formal care were however often made by the dyad prior to consultation with the children. Some carers do not wish to involve their children in the care‐giving process and decisions about the situation of care. In this regard, the carer that is closest to the person with dementia is the most important one in the decision‐making process.

“At the present time, my mother is the one who has the last word since she is the one who lives with dad”. (IT8030, No formal care use, Carer)

## 4 DISCUSSION

For this qualitative study, interviews were conducted with people with dementia and their carers, to explore their attitudes and experiences concerning access to formal care. The results indicate a complex interplay of factors in the process of finding access to care including personal, diagnosis‐related, system‐related and relational factors.

### 4.1 Theme 1: Conditions to enhance access

Concerning personal factors, it is helpful to have an open attitude towards formal care. An open attitude is characterised by absence of shame towards service use and by being open to receive help. In addition, it is helpful to feel that it is justified to make use of formal care services. This finding supports previous research, where stigmatic beliefs and feelings of shame regarding using services were reported as potential barriers (Werner, Goldstein, Karpas, Chan, & Lai, 2014). In the current sample, few people reported a reluctance to use services as it would reflect badly on their ability to care, which might, however, have been due to our sample. Those who are willing to participate in research and share their views are less likely to express shame. Overall, it should be clarified to potential care users that there are a wide range of services and that some can be geared towards the earlier post‐diagnostic stages (these enhance independence and autonomy) while others are more suited to later stages of the condition and if introduced too early, they could create excess disability. In addition, governments and local institutions should pay attention to reducing stigma by increasing awareness with, for example, awareness campaigns and promotion of dementia‐friendly communities.

In most countries, interviewees reported that it is helpful to have a dementia diagnosis while trying to access care. However, in PT, a few carers reported that a diagnosis might also impede access, and it was sometimes concealed while applying for care. This specific barrier may be related to a lack of dementia‐specific community formal services, and of staff that is trained specifically for dementia care.

The country of residence therefore determines if a dementia diagnosis is necessary for accessing care. Nevertheless, the majority of participants reported that having a diagnosis does provide an incentive to seek help.

A number of geographical differences were found between countries. In the more northern countries where care is well in place already, the preference for individualised tailor‐made care was expressed. This contrasts with the situation of the southern countries, where dementia‐specific and easy accessible care is a priority. In addition to that, there are waiting lists, for example in PT.

The results showed in all countries that having a key contact person to guide and support those living with dementia could be very helpful. This supports previous research, where a specific contact person was identified as a marker of best practice (Karlsson et al., 2015; Jansen et al., 2009; Stephan et al., 2018, under review). Another crucial element to optimise access to care is to receive adequate information about dementia and about available resources immediately after the diagnosis. This has also been well established in previous research: providing information post‐diagnosis can delay institutionalisation (Karlsson et al., 2015) and serves as a facilitator in help‐seeking (Werner et al., 2014) (Greenwood & Smith, 2015). Families should also be made aware of any financial assistance that may be available in each country, as expected high costs can serve as a barrier in decision‐making about care.

Carers advised that it was important to be pro‐active in looking for information and services. Being more pro‐active might however be more difficult for the current older generation, as (online) information may be difficult to access for them. Participants expressed the wish that healthcare professionals should have dementia‐specific knowledge and skills. Jansen et al. found that home‐care providers themselves reported the importance of dementia‐training and certificates, as this would lead to higher quality care and higher retention rates of personnel (Jansen et al., 2009). If healthcare professionals do not possess the necessary skills or knowledge, it is important to refer adequately.

We found that it is important to involve the social network, as it can play an important role in assisting with care tasks. Previous research has shown that informal carers often feel reluctant to ask their social network for support (Dam, Boots, van Boxtel, Verhey, & de Vugt, 2017). Healthcare professionals should pay attention to help carers to motivate and mobilise their social network and decrease barriers to ask for support, for example through family meetings. Furthermore, research emphasises the importance of social interaction in relation to (prevention of) dementia (Kuiper et al., 2015).

### 4.2 Theme 2: Decision‐making

Concerning decision‐making, we found that involvement of others beyond the immediate dyad was helpful in some instances, but was not a major influence. Children often play an encouraging role, trying to persuade the parents to take up services. Across all countries, the person closest to the person with dementia is the most important one in decision‐making; this person is often the partner or spouse. Decision‐making is often gradually taken over, from everyday small decisions to major decisions concerning for example service use (Samsi & Manthorpe, 2013).

As cognitive functioning decreases, there is a shift for informal carers from supported or shared decision‐making to substitute decision‐making (Fetherstonhaugh, Rayner, & Tarzia, 2016): this is a gradual process. A phenomenological study found that all participating dyads shifted to a state of substitute decision‐making, but in most cases they tried to maintain the autonomy of the person with dementia for as long as possible (Samsi & Manthorpe, 2013). Since this is a complex process that should be attended with care, healthcare professionals should be equipped to support the person closest to the person with dementia and to be a mediator between the carer and the person with dementia.

The main outcome of a previous focus group study was that needs of the person with dementia and the informal care‐givers should be balanced, a so‐called dyadic focus. On the care‐givers' side there is a need for support and knowledge, while on the person with dementia's side one should take the need for integrity into account (Karlsson et al., 2015). It is important that people with dementia retain a sense of autonomy by being able to participate in everyday decisions (Fetherstonhaugh et al., 2016). This can for example be established with shared decision‐making, where all individual needs are taken into account. This is in line with previous research by Boyle et al. (Boyle, 2014) which states that people with dementia may not have the full capacity to make all decisions, but they do still have the ability to make other types of decisions for example related to domestic care and social life.

There are some methodological strengths and limitations to be discussed. Within the Actifcare cohort, we were able to interview an international and diverse group, which enabled us to compare experiences across countries. Another strength of this design is that in some of the interviews the person with dementia was also included, in addition to the informal care‐giver. The interview questions were based on the outcomes of previously held focus groups, to ensure current relevance of each topic. One of the methodological limitations is that it was not possible to interview until data saturation was reached. A sample size of 10 per country was defined 'a priori' to take into account time restrictions and schedules. In addition, we were not able to interview the dyad both together and separately in all cases, and we do feel that in some cases participants did not speak freely while being interviewed together. Given our methodology, results are not generalisable. Another limitation is selection bias: people who refuse to use services are not likely to take part in a study concerning service us, as they prefer no interference at all. Due to this, we miss information about the experiences of those refusing formal care.

### 4.3 Clinical implications

These results have several clinical implications. Healthcare professionals should attend to factors that are modifiable during the process of finding access to care. In addition, tailored advice should be given, and healthcare professionals should act as a mediator in dyads' decision‐making process, and support them with techniques such as motivational interviewing and family meetings. These findings, as well as findings from the other Actifcare work packages (see Figure 1), informed the development of best practice recommendations (Røsvik et al., 2018), these can be found at our website (http://www.actifcare.eu).

## 5 CONCLUSION

Based on the outcomes of 85 in‐depth interviews across Europe, we summarise the factors for optimal access to care in a pathway (Figure 2). The results indicate that there are personal factors, diagnosis‐related factors and system‐related factors involved in finding optimal access to care. There are substantial differences between countries regarding waiting lists, disclosure of diagnosis, dementia‐specific services and financial compensations for service use. In addition, culture plays a role as care‐giving tends to be seen as a moral obligation in southern countries.

On the basis of these and other Actifcare study outcomes, best practice recommendations have been developed (http://www.actifcare.eu). The current study provided in‐depth insight into people's attitudes and experiences barriers and facilitators with regard to access to dementia care that can help healthcare professionals and policy makers to optimise timely access to care across Europe.

## ETHICS APPROVAL

All individual countries have applied for medical ethical approval in their own country. Ethical consideration differs between countries: Medische‐ ethische toetsingscommissie (NL), Wales Research Ethics Committee 5, Bangor (UK), Ethics committee of the Medical Faculty, Martin Luther University Halle‐Wittenberg (DE), Regional committee for medical and health research ethics, South‐East B (NO), the Regional Ethics Review Board (SW), Dublin City University Research Ethics Committee (IE), Ethics Committee of the Nova Medical School, Ethics Committee of Centro Hospitalar de Lisboa Ocidental, Ethics Committee of ARSLVT, Ethics Committee of ARSA, Comissão Nacional de Protecção de Dados (PT). Comitato Etico, IRCCS San Giovanni di Dio‐ Fatebenefratelli (IT). All participating NHS sites in the UK received permission to perform the study. The study protocol complies with the Medical Research Involving Human Subjects Act and codes on “good use” of clinical data.

## DISCLOSURE OF INTEREST

No conflicts of interest have been declared.
