# Supplementary material for: Optimizing access to and use of formal dementia care: Qualitative findings from the European Actifcare study
Source: Health Soc Care Community. 2019 Jul 10;27(5):e814–23. doi: 10.1111/hsc.12804 (PMC6851600; doi:10.1111/hsc.12804)
Supplement: Supplementary file 1 [file HSC-27-e814-s001.docx]

# Appendix 1: Interview guide

The following themes are based on the findings of the focus group interviews. They have been developed by the German working group.

**A: Receiving the diagnosis**

Introduction: you received a dementia diagnosis: that must have been an emotional and difficult time. We are interested if this diagnosis has ‘helped’ you as well, namely with finding access to care.

| Has/ have initiated formal care | Has/ have NOT initiated formal care |
| --- | --- |
| *Main question*  *A1. Did receiving a dementia diagnosis influence the process of access to care?*  *Questions (for further exploration)*  *A1.1 Was disclosure/receiving of the diagnosis followed by guidance or support?  A1.2 Were you satisfied or dissatisfied with this situation (i.e. with (not) receiving guidance or support after the diagnosis) ?(please elaborate).   A1.3 Do you think a dementia diagnosis is necessary for access to formal dementia care?* | *Questions (for further exploration)*  *A1.1 Was disclosure/receiving of the diagnosis followed by guidance or support?  A1.2 Were you satisfied or dissatisfied with this situation (i.e. with (not) receiving guidance or support after the diagnosis) ?(please elaborate).   A1.3 Do you think a dementia diagnosis is necessary for access to formal dementia care?* |

**B (and C?): Attitude towards formal care (meaning of formal care)**

Introduction: we are interested in your experiences with regard to asking for and receiving care.

| Has/ have initiated formal care | Has/ have NOT initiated formal care |
| --- | --- |
| *Main question*  *B1. What is your attitude towards receiving (formal) dementia care or asking for it? (we are interested in both opinions of pwd and carer).*  *B1.1 Do you feel embarrassed/have scruples or is it easy for you?*  *Questions (for further exploration)*  *B2.1 How do you feel that you (pwd) / your husband/wife is using …… (fill out this information based on FU2)? Explore opinions / reasons!*  *B2.2 How do you feel about other people knowing that you make use of formal care?* | *Main question*  *B1. What is your attitude towards receiving (formal) dementia care or asking for it? (we are interested in both opinions of pwd and carer).*  *B2.3 Have you considered a time in the future where you would start using care, (and what would make the difference?) What type of circumstances can you imagine which would lead to service use in the future?* |
| Has/ have initiated formal care | Has/ have NOT initiated formal care |
| *C1.1 Does it influence your ability to stay independent?*  *Questions (for further exploration)*  *C1.1 Did you experience formal care as a limitation or as an enhancement of your independence?* | *C1.1 Would receiving services influence your ability to stay independent?* |

**D: Exchanging views within the family - Influence on joint decision making?**

| Has/ have initiated formal care | Has/ have NOT initiated formal care |
| --- | --- |
| *Main question*  *D1. Do you (carer and PwD) share similar views on initiating or accepting help?(explore what these views are)   Questions (for further exploration)*  *D1.1 Is there someone else in the family (e.g. children) who play a role in this decision making?*  *D1.2 Is this a topic that you can easily discuss with each other? (explore!)  What does a conversation like this look like, is it emotional, does it take long, who takes the lead, do you need to make an effort to convince the other person?  D1.3 How do you deal with dissimilarities?* | *Main question*  *D1. Do you (carer and PwD) share similar views on initiating or accepting help?(explore what these views are)   Questions (for further exploration)*  *D1.1 Is there someone else in the family (e.g. children) who play a role in this decision making?*  *D1.2 Is this a topic that you can easily discuss with each other? (explore!)  What does a conversation like this look like, is it emotional, does it take long, who takes the lead, do you need to make an effort to convince the other person?  D1.3 How do you deal with dissimilarities?*  *D1.4 Was it a joint decision to start using formal care?* |

**E: Cooperation with healthcare professionals (focus on facilitating aspects) joint decision making**

| Has/ have initiated formal care | Has/ have NOT initiated formal care |
| --- | --- |
| *Main question*  *E1. How do you experience the cooperation with your health care professionals in the process of access to care? (explore!)  Questions (for further exploration)*  *E1.1 What difficulties did you experience? Did you miss anything?*  *E1.2 What helped you or would have helped you in this process?*  *E1.3 Is there any advice that you would give to the professionals regarding the process of (finding) access to care?*  *E1.4 Is there any advice you could give to other carers and people with dementia regarding the process of (finding) access to care?* | *E1.3 Is there any advice that you would give to the professionals regarding the process of (finding) access to care?*  *E1.4 Is there any advice you could give to other carers and people with dementia regarding the process of (finding) access to care?* |
